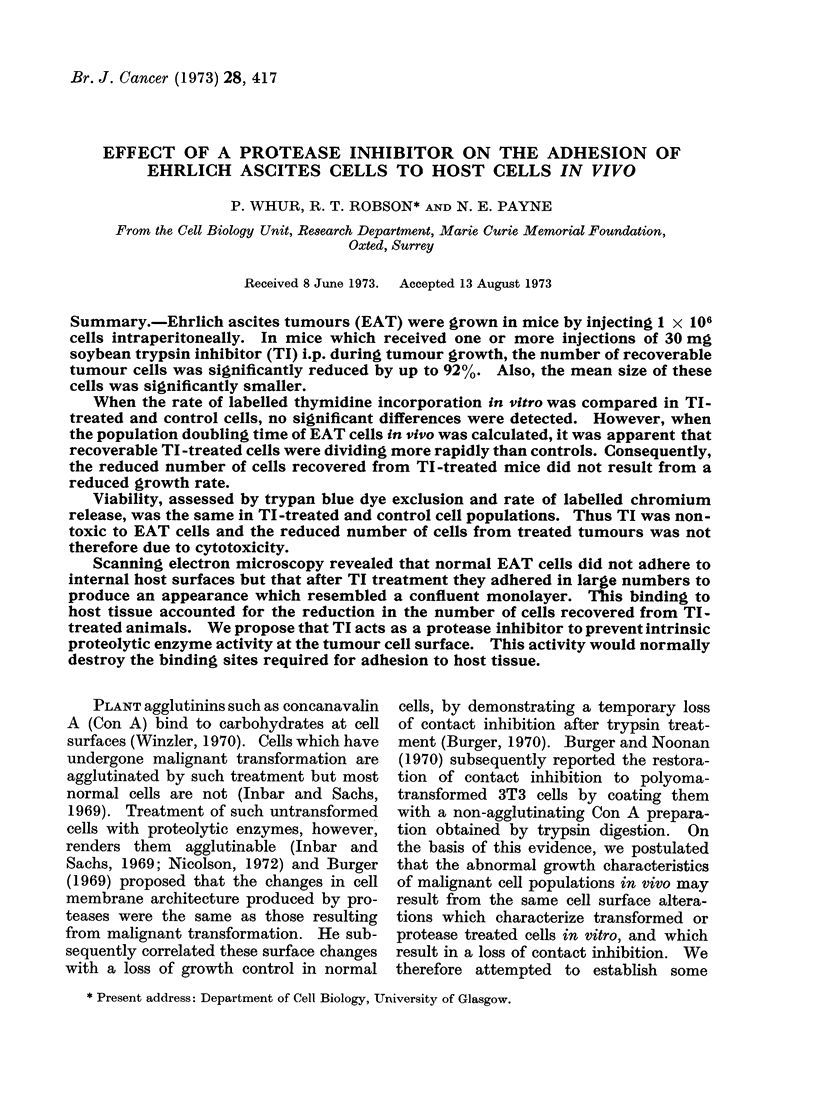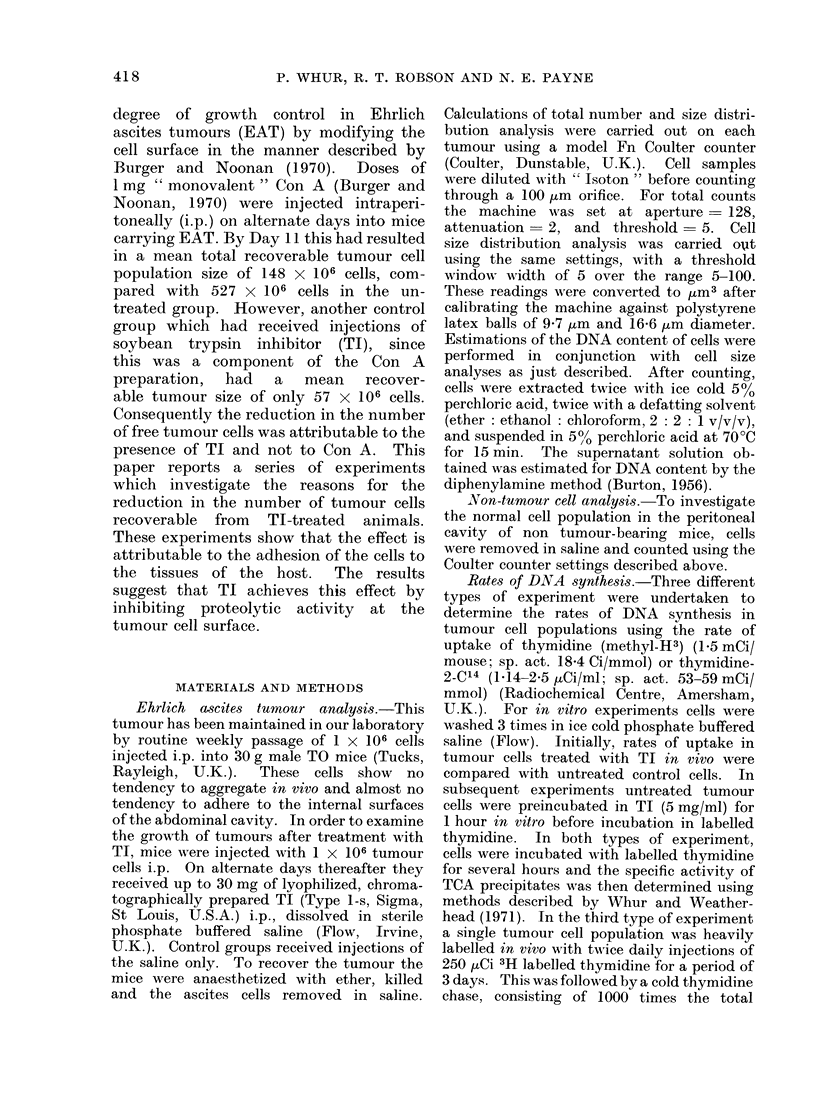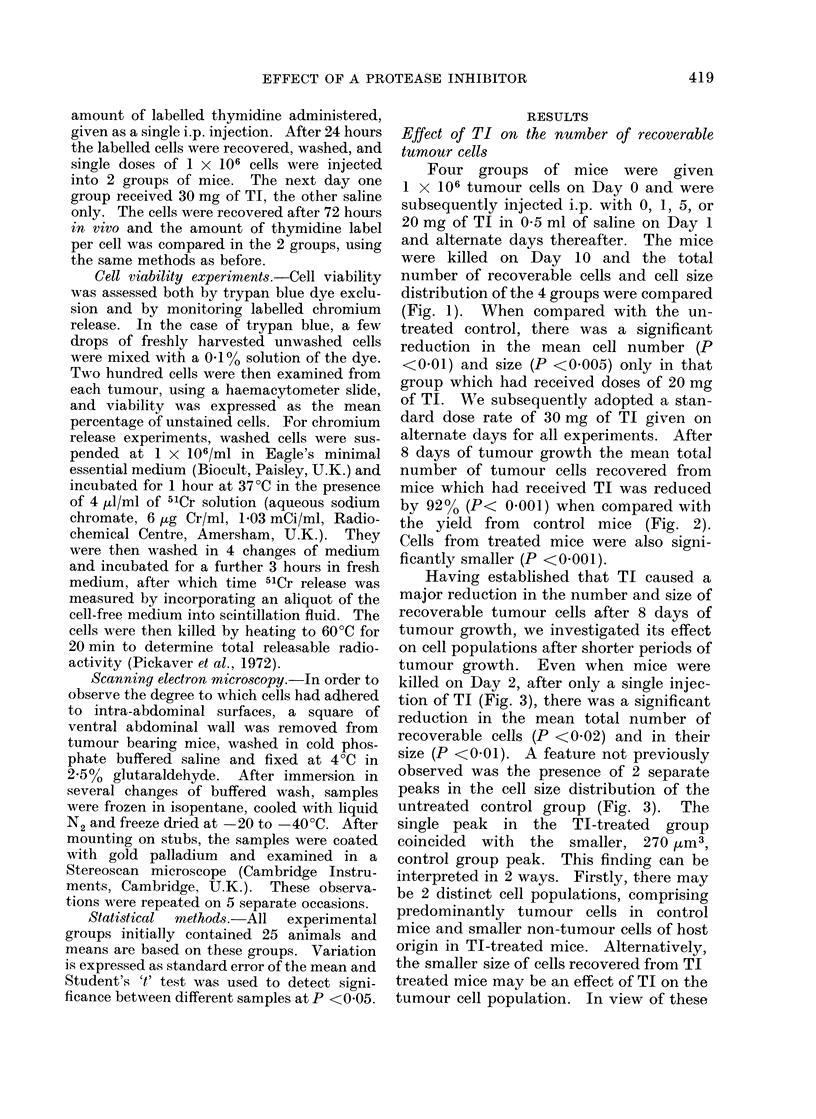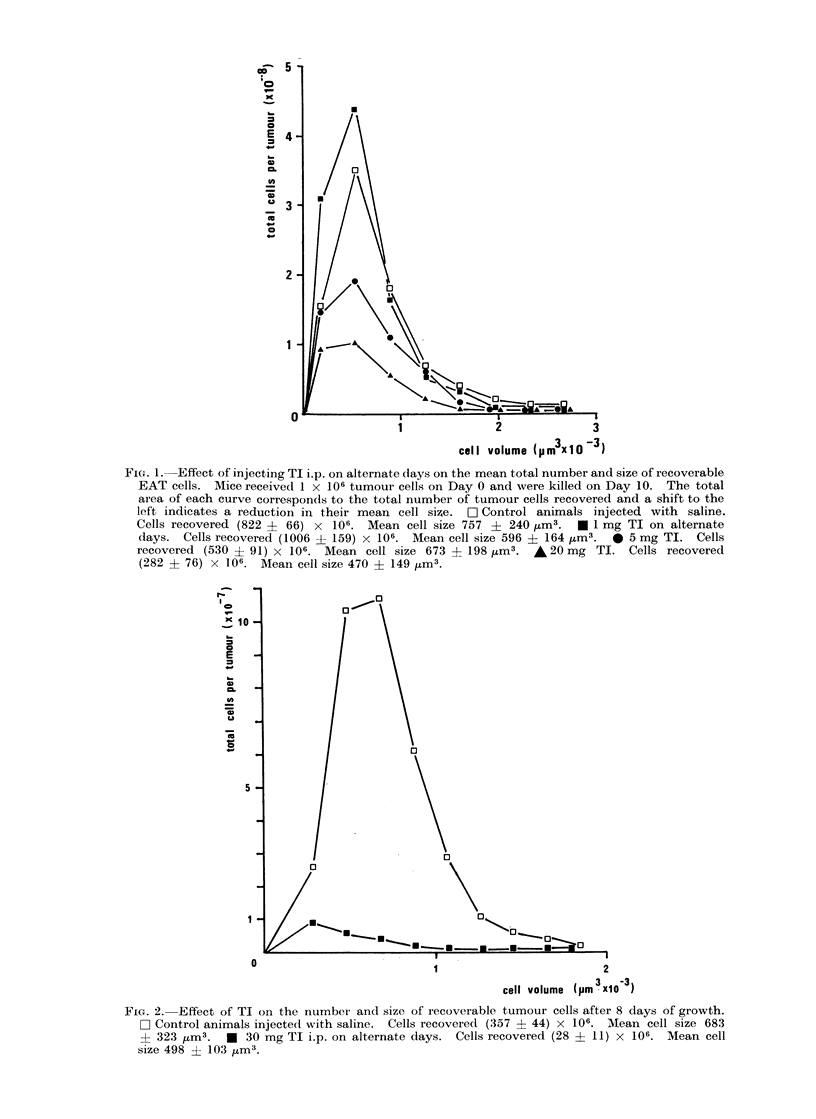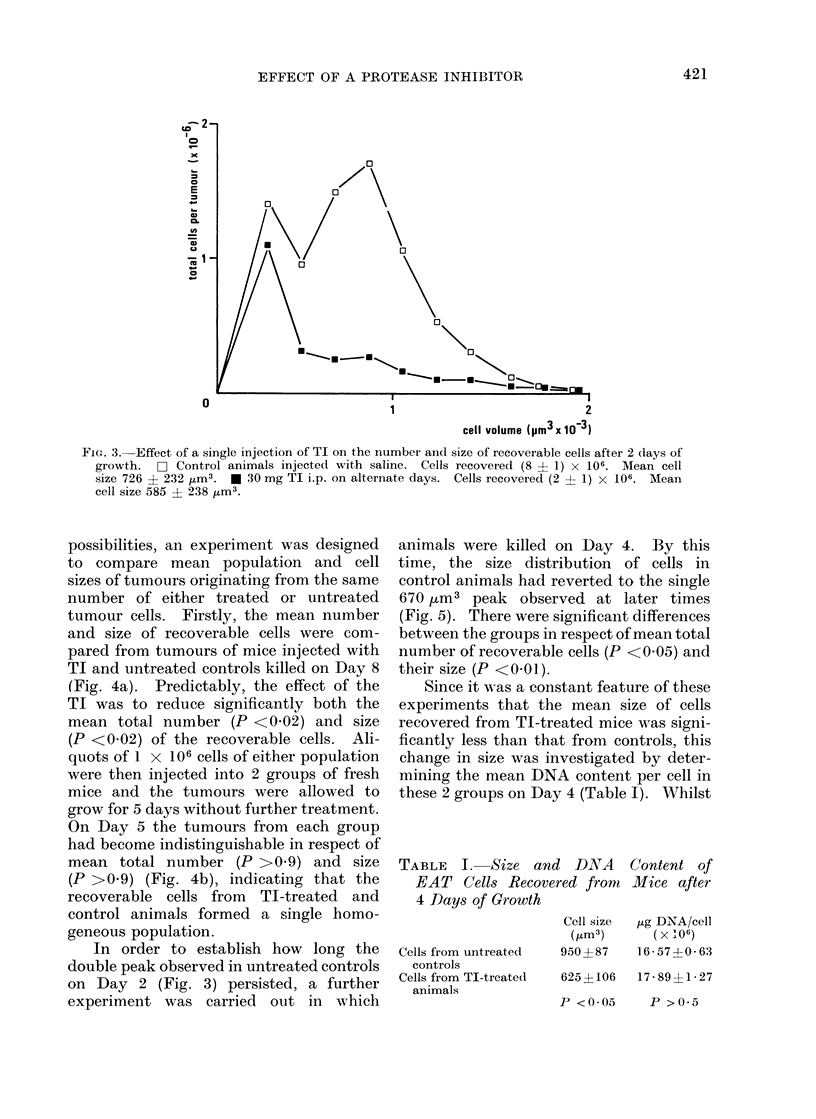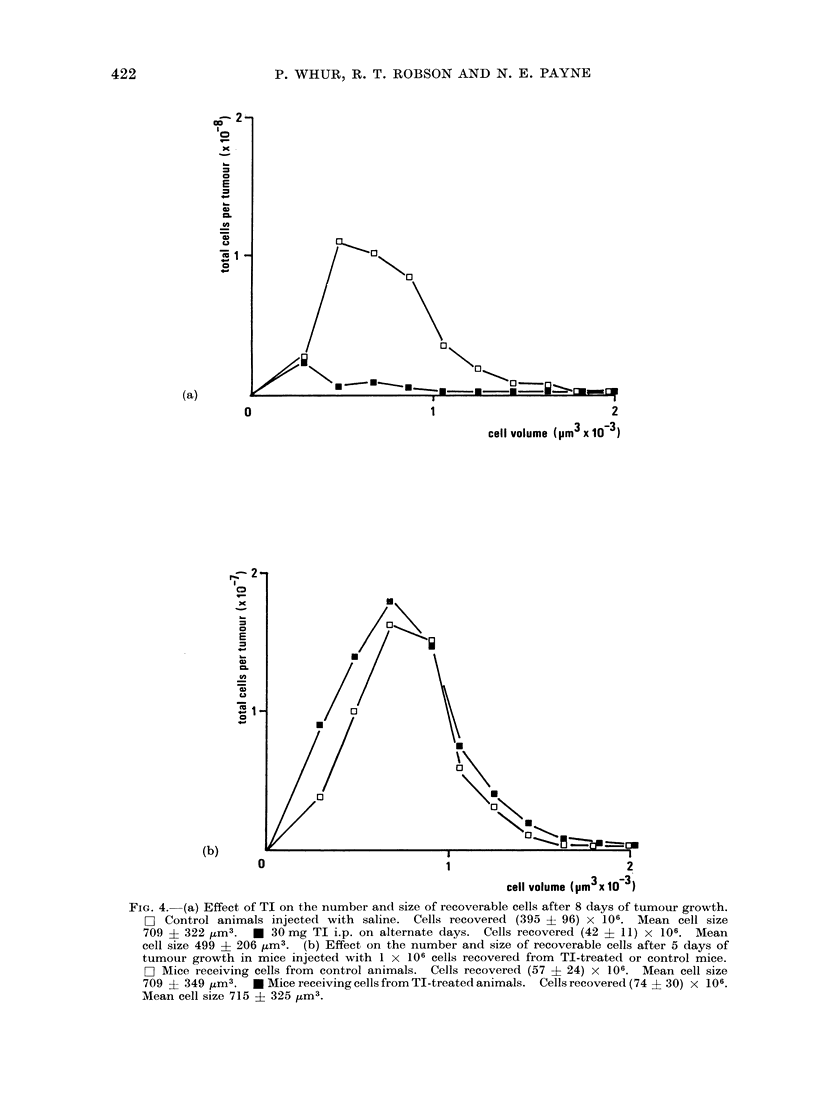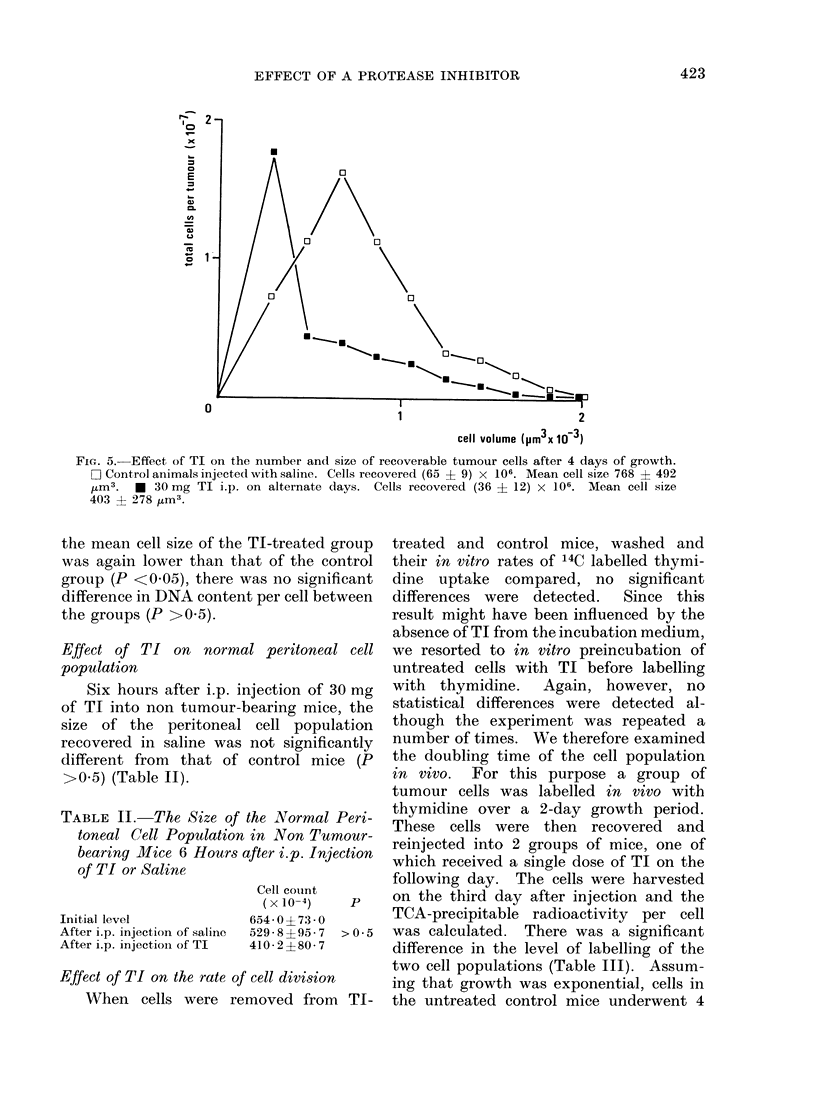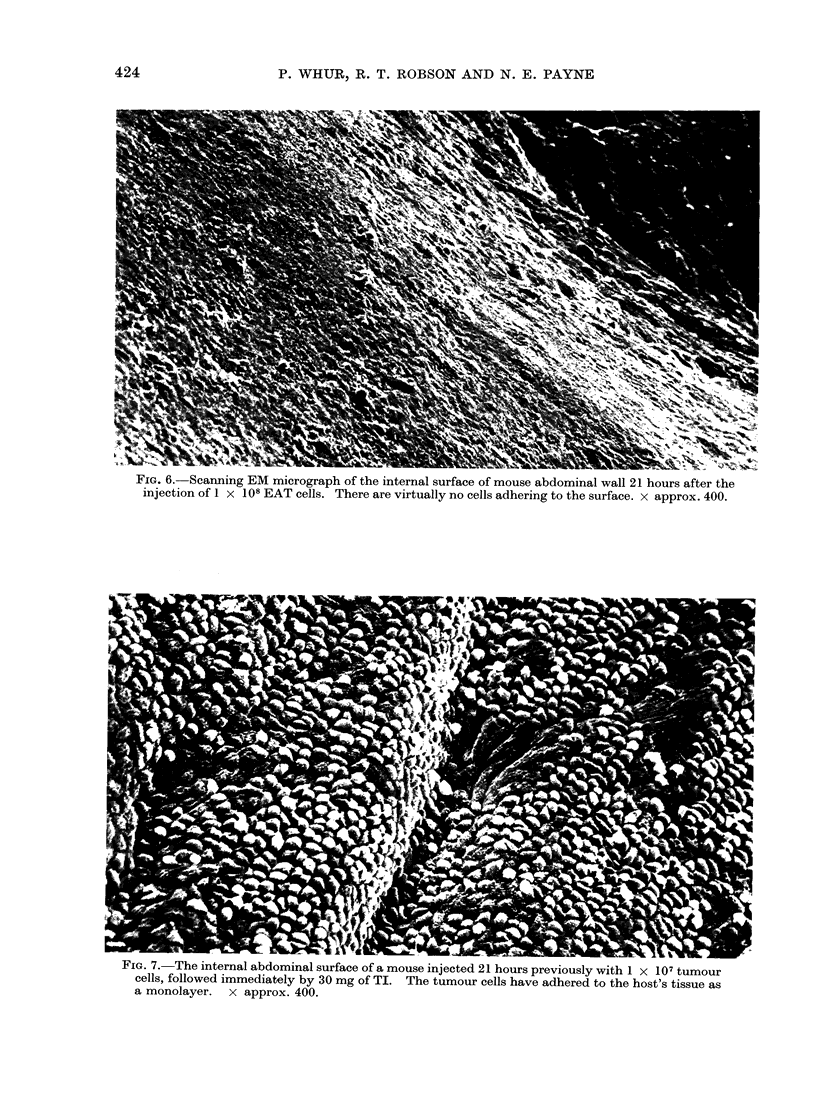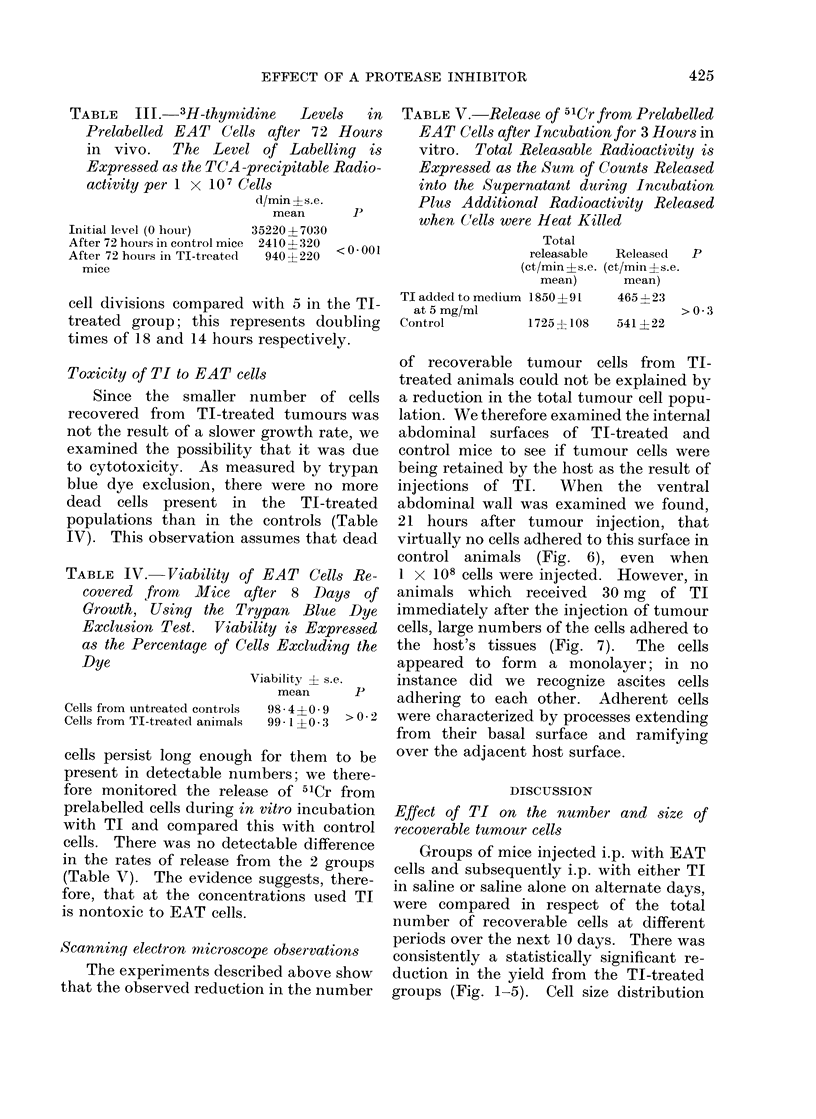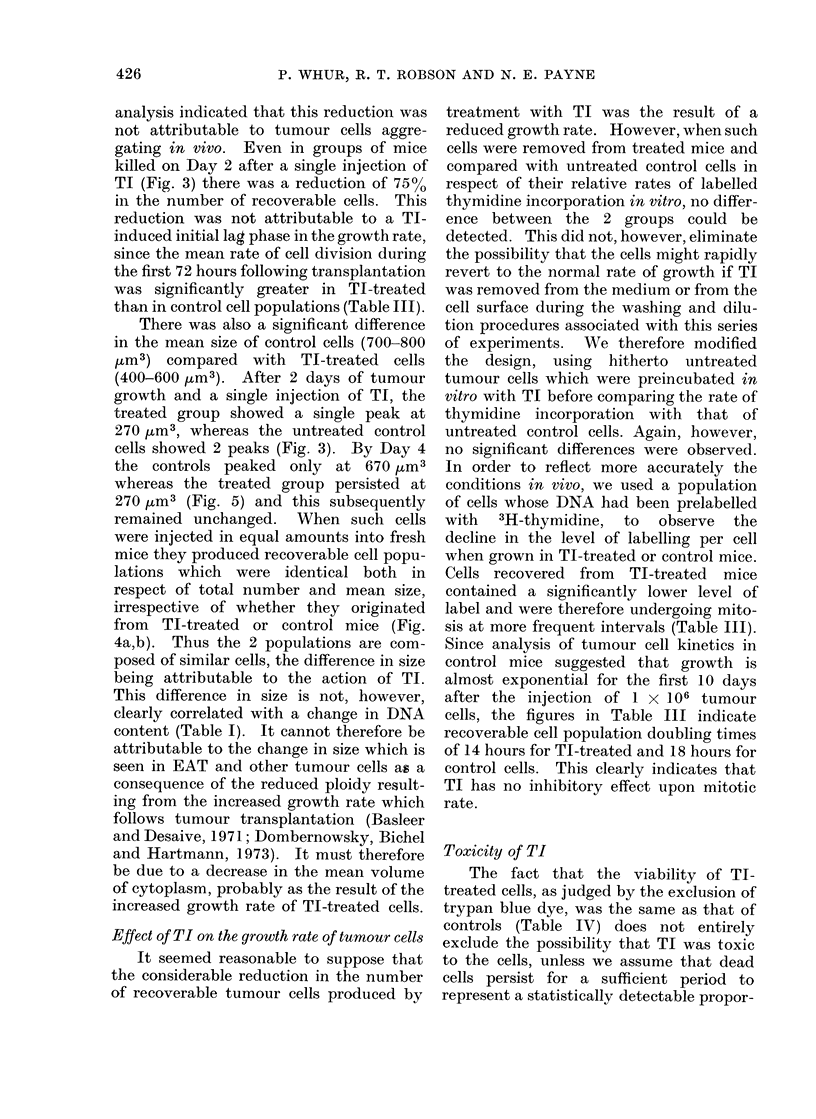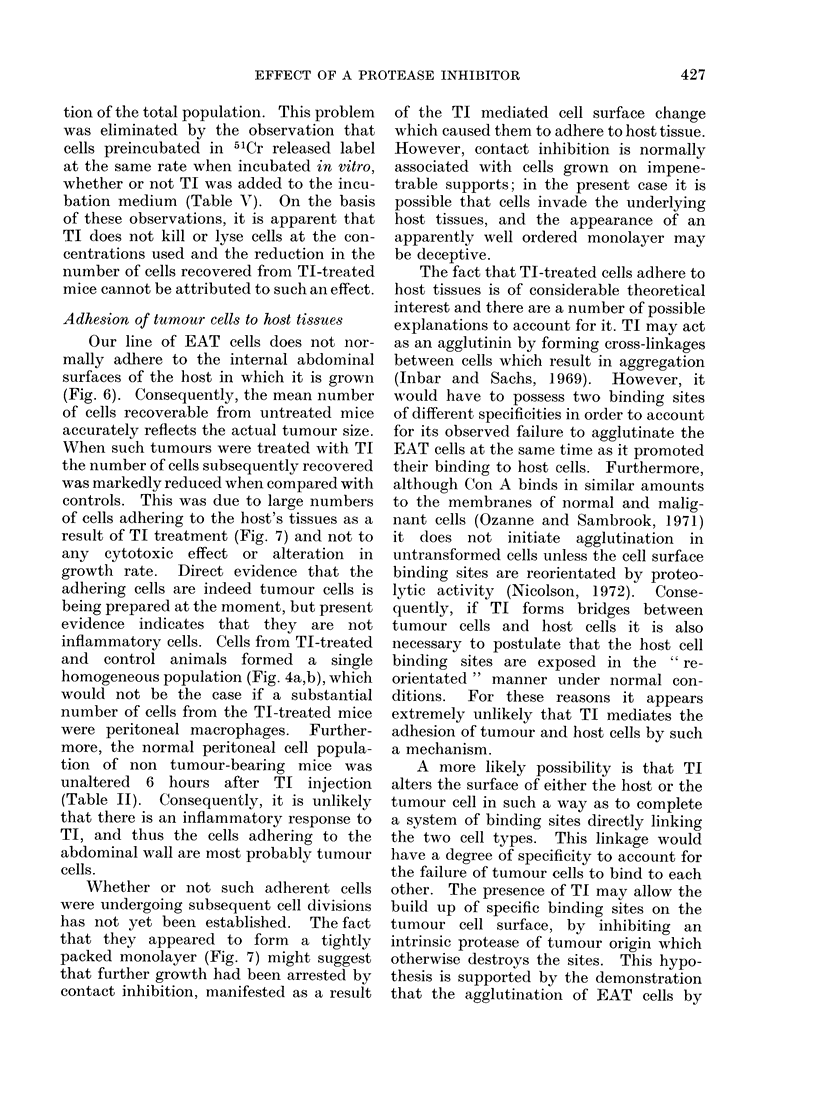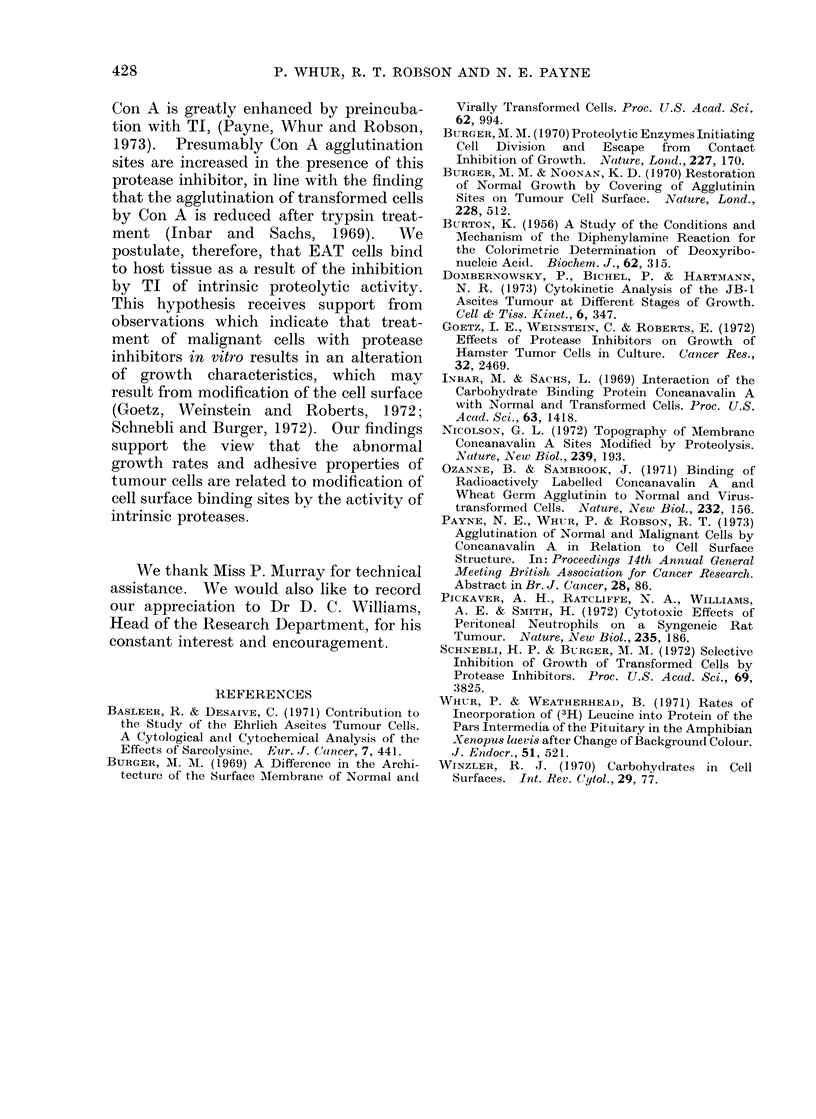# Effect of a Protease Inhibitor on the Adhesion of Ehrlich Ascites Cells to Host Cells in vivo

**DOI:** 10.1038/bjc.1973.167

**Published:** 1973-11

**Authors:** P. Whur, R. T. Robson, N. E. Payne

## Abstract

**Images:**


*Br. J. Cancer* (1973) **28**, 417

### EFFECT OF A PROTEASE INHIBITOR ON THE ADHESION OF EHRLICH ASCITES CELLS TO HOST CELLS *IN VIVO*

P. WHUR, R. T. ROBSON* AND N. E. PAYNE

*From the Cell Biology Unit, Research Department, Marie Curie Memorial Foundation, Oxted, Surrey*

Received 8 June 1973. Accepted 13 August 1973

**Summary.—Ehrlich ascites tumours (EAT) were grown in mice by injecting 1 $\times$ $10^6$ cells intraperitoneally. In mice which received one or more injections of 30 mg soybean trypsin inhibitor (TI) i.p. during tumour growth, the number of recoverable tumour cells was significantly reduced by up to 92%. Also, the mean size of these cells was significantly smaller.**

**When the rate of labelled thymidine incorporation *in vitro* was compared in TI-treated and control cells, no significant differences were detected. However, when the population doubling time of EAT cells *in vivo* was calculated, it was apparent that recoverable TI-treated cells were dividing more rapidly than controls. Consequently, the reduced number of cells recovered from TI-treated mice did not result from a reduced growth rate.**

**Viability, assessed by trypan blue dye exclusion and rate of labelled chromium release, was the same in TI-treated and control cell populations. Thus TI was non-toxic to EAT cells and the reduced number of cells from treated tumours was not therefore due to cytotoxicity.**

**Scanning electron microscopy revealed that normal EAT cells did not adhere to internal host surfaces but that after TI treatment they adhered in large numbers to produce an appearance which resembled a confluent monolayer. This binding to host tissue accounted for the reduction in the number of cells recovered from TI-treated animals. We propose that TI acts as a protease inhibitor to prevent intrinsic proteolytic enzyme activity at the tumour cell surface. This activity would normally destroy the binding sites required for adhesion to host tissue.**

PLANT agglutinins such as concanavalin A (Con A) bind to carbohydrates at cell surfaces (Winzler, 1970). Cells which have undergone malignant transformation are agglutinated by such treatment but most normal cells are not (Inbar and Sachs, 1969). Treatment of such untransformed cells with proteolytic enzymes, however, renders them agglutinable (Inbar and Sachs, 1969; Nicolson, 1972) and Burger (1969) proposed that the changes in cell membrane architecture produced by proteases were the same as those resulting from malignant transformation. He subsequently correlated these surface changes with a loss of growth control in normal cells, by demonstrating a temporary loss of contact inhibition after trypsin treatment (Burger, 1970). Burger and Noonan (1970) subsequently reported the restoration of contact inhibition to polyoma-transformed 3T3 cells by coating them with a non-agglutinating Con A preparation obtained by trypsin digestion. On the basis of this evidence, we postulated that the abnormal growth characteristics of malignant cell populations *in vivo* may result from the same cell surface alterations which characterize transformed or protease treated cells *in vitro*, and which result in a loss of contact inhibition. We therefore attempted to establish some

* Present address: Department of Cell Biology, University of Glasgow.

degree of growth control in Ehrlich ascites tumours (EAT) by modifying the cell surface in the manner described by Burger and Noonan (1970). Doses of 1 mg " monovalent " Con A (Burger and Noonan, 1970) were injected intraperitoneally (i.p.) on alternate days into mice carrying EAT. By Day 11 this had resulted in a mean total recoverable tumour cell population size of $148 \times 10^6$ cells, compared with $527 \times 10^6$ cells in the untreated group. However, another control group which had received injections of soybean trypsin inhibitor (TI), since this was a component of the Con A preparation, had a mean recoverable tumour size of only $57 \times 10^6$ cells. Consequently the reduction in the number of free tumour cells was attributable to the presence of TI and not to Con A. This paper reports a series of experiments which investigate the reasons for the reduction in the number of tumour cells recoverable from TI-treated animals. These experiments show that the effect is attributable to the adhesion of the cells to the tissues of the host. The results suggest that TI achieves this effect by inhibiting proteolytic activity at the tumour cell surface.

### MATERIALS AND METHODS

*Ehrlich ascites tumour analysis.*—This tumour has been maintained in our laboratory by routine weekly passage of $1 \times 10^6$ cells injected i.p. into 30 g male TO mice (Tucks, Rayleigh, U.K.). These cells show no tendency to aggregate *in vivo* and almost no tendency to adhere to the internal surfaces of the abdominal cavity. In order to examine the growth of tumours after treatment with TI, mice were injected with $1 \times 10^6$ tumour cells i.p. On alternate days thereafter they received up to 30 mg of lyophilized, chromatographically prepared TI (Type 1-s, Sigma, St Louis, U.S.A.) i.p., dissolved in sterile phosphate buffered saline (Flow, Irvine, U.K.). Control groups received injections of the saline only. To recover the tumour the mice were anaesthetized with ether, killed and the ascites cells removed in saline. Calculations of total number and size distribution analysis were carried out on each tumour using a model Fn Coulter counter (Coulter, Dunstable, U.K.). Cell samples were diluted with " Isoton " before counting through a 100 $\mu$m orifice. For total counts the machine was set at aperture = 128, attenuation = 2, and threshold = 5. Cell size distribution analysis was carried out using the same settings, with a threshold window width of 5 over the range 5–100. These readings were converted to $\mu m^3$ after calibrating the machine against polystyrene latex balls of 9·7 $\mu$m and 16·6 $\mu$m diameter. Estimations of the DNA content of cells were performed in conjunction with cell size analyses as just described. After counting, cells were extracted twice with ice cold 5% perchloric acid, twice with a defatting solvent (ether : ethanol : chloroform, 2 : 2 : 1 v/v/v), and suspended in 5% perchloric acid at 70°C for 15 min. The supernatant solution obtained was estimated for DNA content by the diphenylamine method (Burton, 1956).

*Non-tumour cell analysis.*—To investigate the normal cell population in the peritoneal cavity of non tumour-bearing mice, cells were removed in saline and counted using the Coulter counter settings described above.

*Rates of DNA synthesis.*—Three different types of experiment were undertaken to determine the rates of DNA synthesis in tumour cell populations using the rate of uptake of thymidine (methyl-$H^3$) (1·5 mCi/mouse; sp. act. 18·4 Ci/mmol) or thymidine-2-$C^{14}$ (1·14–2·5 $\mu$Ci/ml; sp. act. 53–59 mCi/mmol) (Radiochemical Centre, Amersham, U.K.). For *in vitro* experiments cells were washed 3 times in ice cold phosphate buffered saline (Flow). Initially, rates of uptake in tumour cells treated with TI *in vivo* were compared with untreated control cells. In subsequent experiments untreated tumour cells were preincubated in TI (5 mg/ml) for 1 hour *in vitro* before incubation in labelled thymidine. In both types of experiment, cells were incubated with labelled thymidine for several hours and the specific activity of TCA precipitates was then determined using methods described by Whur and Weatherhead (1971). In the third type of experiment a single tumour cell population was heavily labelled *in vivo* with twice daily injections of 250 $\mu$Ci $^3$H labelled thymidine for a period of 3 days. This was followed by a cold thymidine chase, consisting of 1000 times the total amount of labelled thymidine administered, given as a single i.p. injection. After 24 hours the labelled cells were recovered, washed, and single doses of $1 \times 10^6$ cells were injected into 2 groups of mice. The next day one group received 30 mg of TI, the other saline only. The cells were recovered after 72 hours *in vivo* and the amount of thymidine label per cell was compared in the 2 groups, using the same methods as before.

*Cell viability experiments.*—Cell viability was assessed both by trypan blue dye exclusion and by monitoring labelled chromium release. In the case of trypan blue, a few drops of freshly harvested unwashed cells were mixed with a 0·1% solution of the dye. Two hundred cells were then examined from each tumour, using a haemacytometer slide, and viability was expressed as the mean percentage of unstained cells. For chromium release experiments, washed cells were suspended at $1 \times 10^6$/ml in Eagle's minimal essential medium (Biocult, Paisley, U.K.) and incubated for 1 hour at 37°C in the presence of 4 $\mu$l/ml of $^{51}$Cr solution (aqueous sodium chromate, 6 $\mu$g Cr/ml, 1·03 mCi/ml, Radiochemical Centre, Amersham, U.K.). They were then washed in 4 changes of medium and incubated for a further 3 hours in fresh medium, after which time $^{51}$Cr release was measured by incorporating an aliquot of the cell-free medium into scintillation fluid. The cells were then killed by heating to 60°C for 20 min to determine total releasable radioactivity (Pickaver *et al.*, 1972).

*Scanning electron microscopy.*—In order to observe the degree to which cells had adhered to intra-abdominal surfaces, a square of ventral abdominal wall was removed from tumour bearing mice, washed in cold phosphate buffered saline and fixed at 4°C in 2·5% glutaraldehyde. After immersion in several changes of buffered wash, samples were frozen in isopentane, cooled with liquid $N_2$ and freeze dried at −20 to −40°C. After mounting on stubs, the samples were coated with gold palladium and examined in a Stereoscan microscope (Cambridge Instruments, Cambridge, U.K.). These observations were repeated on 5 separate occasions.

*Statistical methods.*—All experimental groups initially contained 25 animals and means are based on these groups. Variation is expressed as standard error of the mean and Student's '*t*' test was used to detect significance between different samples at $P < 0{\cdot}05$.

### RESULTS

### *Effect of TI on the number of recoverable tumour cells*

Four groups of mice were given $1 \times 10^6$ tumour cells on Day 0 and were subsequently injected i.p. with 0, 1, 5, or 20 mg of TI in 0·5 ml of saline on Day 1 and alternate days thereafter. The mice were killed on Day 10 and the total number of recoverable cells and cell size distribution of the 4 groups were compared (Fig. 1). When compared with the untreated control, there was a significant reduction in the mean cell number ($P < 0{\cdot}01$) and size ($P < 0{\cdot}005$) only in that group which had received doses of 20 mg of TI. We subsequently adopted a standard dose rate of 30 mg of TI given on alternate days for all experiments. After 8 days of tumour growth the mean total number of tumour cells recovered from mice which had received TI was reduced by 92% ($P < 0{\cdot}001$) when compared with the yield from control mice (Fig. 2). Cells from treated mice were also significantly smaller ($P < 0{\cdot}001$).

Having established that TI caused a major reduction in the number and size of recoverable tumour cells after 8 days of tumour growth, we investigated its effect on cell populations after shorter periods of tumour growth. Even when mice were killed on Day 2, after only a single injection of TI (Fig. 3), there was a significant reduction in the mean total number of recoverable cells ($P < 0{\cdot}02$) and in their size ($P < 0{\cdot}01$). A feature not previously observed was the presence of 2 separate peaks in the cell size distribution of the untreated control group (Fig. 3). The single peak in the TI-treated group coincided with the smaller, 270 $\mu m^3$, control group peak. This finding can be interpreted in 2 ways. Firstly, there may be 2 distinct cell populations, comprising predominantly tumour cells in control mice and smaller non-tumour cells of host origin in TI-treated mice. Alternatively, the smaller size of cells recovered from TI treated mice may be an effect of TI on the tumour cell population. In view of these

Fig. 1.—Effect of injecting TI i.p. on alternate days on the mean total number and size of recoverable EAT cells. Mice received 1 × $10^6$ tumour cells on Day 0 and were killed on Day 10. The total area of each curve corresponds to the total number of tumour cells recovered and a shift to the left indicates a reduction in their mean cell size. □ Control animals injected with saline. Cells recovered (822 ± 66) × $10^6$. Mean cell size 757 ± 240 $\mu m^3$. ■ 1 mg TI on alternate days. Cells recovered (1006 ± 159) × $10^6$. Mean cell size 596 ± 164 $\mu m^3$. ● 5 mg TI. Cells recovered (530 ± 91) × $10^6$. Mean cell size 673 ± 198 $\mu m^3$. ▲ 20 mg TI. Cells recovered (282 ± 76) × $10^6$. Mean cell size 470 ± 149 $\mu m^3$.

Fig. 2.—Effect of TI on the number and size of recoverable tumour cells after 8 days of growth. □ Control animals injected with saline. Cells recovered (357 ± 44) × $10^6$. Mean cell size 683 ± 323 $\mu m^3$. ■ 30 mg TI i.p. on alternate days. Cells recovered (28 ± 11) × $10^6$. Mean cell size 498 ± 103 $\mu m^3$.

FIG. 3.—Effect of a single injection of TI on the number and size of recoverable cells after 2 days of growth. □ Control animals injected with saline. Cells recovered $(8 \pm 1) \times 10^6$. Mean cell size $726 \pm 232$ μm$^3$. ■ 30 mg TI i.p. on alternate days. Cells recovered $(2 \pm 1) \times 10^6$. Mean cell size $585 \pm 238$ μm$^3$.

possibilities, an experiment was designed to compare mean population and cell sizes of tumours originating from the same number of either treated or untreated tumour cells. Firstly, the mean number and size of recoverable cells were compared from tumours of mice injected with TI and untreated controls killed on Day 8 (Fig. 4a). Predictably, the effect of the TI was to reduce significantly both the mean total number ($P < 0{\cdot}02$) and size ($P < 0{\cdot}02$) of the recoverable cells. Aliquots of $1 \times 10^6$ cells of either population were then injected into 2 groups of fresh mice and the tumours were allowed to grow for 5 days without further treatment. On Day 5 the tumours from each group had become indistinguishable in respect of mean total number ($P > 0{\cdot}9$) and size ($P > 0{\cdot}9$) (Fig. 4b), indicating that the recoverable cells from TI-treated and control animals formed a single homogeneous population.

In order to establish how long the double peak observed in untreated controls on Day 2 (Fig. 3) persisted, a further experiment was carried out in which animals were killed on Day 4. By this time, the size distribution of cells in control animals had reverted to the single 670 μm$^3$ peak observed at later times (Fig. 5). There were significant differences between the groups in respect of mean total number of recoverable cells ($P < 0{\cdot}05$) and their size ($P < 0{\cdot}01$).

Since it was a constant feature of these experiments that the mean size of cells recovered from TI-treated mice was significantly less than that from controls, this change in size was investigated by determining the mean DNA content per cell in these 2 groups on Day 4 (Table I). Whilst

TABLE I.—*Size and DNA Content of EAT Cells Recovered from Mice after 4 Days of Growth*

| | Cell size (μm$^3$) | μg DNA/cell (×10$^6$) |
|---|---|---|
| Cells from untreated controls | $950 \pm 87$ | $16{\cdot}57 \pm 0{\cdot}63$ |
| Cells from TI-treated animals | $625 \pm 106$ | $17{\cdot}89 \pm 1{\cdot}27$ |
| | $P < 0{\cdot}05$ | $P > 0{\cdot}5$ |

FIG. 4.—(a) Effect of TI on the number and size of recoverable cells after 8 days of tumour growth. □ Control animals injected with saline. Cells recovered $(395 \pm 96) \times 10^6$. Mean cell size $709 \pm 322\ \mu m^3$. ■ 30 mg TI i.p. on alternate days. Cells recovered $(42 \pm 11) \times 10^6$. Mean cell size $499 \pm 206\ \mu m^3$. (b) Effect on the number and size of recoverable cells after 5 days of tumour growth in mice injected with $1 \times 10^6$ cells recovered from TI-treated or control mice. □ Mice receiving cells from control animals. Cells recovered $(57 \pm 24) \times 10^6$. Mean cell size $709 \pm 349\ \mu m^3$. ■ Mice receiving cells from TI-treated animals. Cells recovered $(74 \pm 30) \times 10^6$. Mean cell size $715 \pm 325\ \mu m^3$.

FIG. 5.—Effect of TI on the number and size of recoverable tumour cells after 4 days of growth. □ Control animals injected with saline. Cells recovered (65 ± 9) × $10^6$. Mean cell size 768 ± 492 $\mu m^3$. ■ 30 mg TI i.p. on alternate days. Cells recovered (36 ± 12) × $10^6$. Mean cell size 403 ± 278 $\mu m^3$.

the mean cell size of the TI-treated group was again lower than that of the control group ($P < 0{\cdot}05$), there was no significant difference in DNA content per cell between the groups ($P > 0{\cdot}5$).

*Effect of TI on normal peritoneal cell population*

Six hours after i.p. injection of 30 mg of TI into non tumour-bearing mice, the size of the peritoneal cell population recovered in saline was not significantly different from that of control mice ($P > 0{\cdot}5$) (Table II).

TABLE II.—*The Size of the Normal Peritoneal Cell Population in Non Tumour-bearing Mice 6 Hours after i.p. Injection of TI or Saline*

| | Cell count ($\times 10^{-4}$) | $P$ |
|---|---|---|
| Initial level | 654·0±73·0 | |
| After i.p. injection of saline | 529·8±95·7 | >0·5 |
| After i.p. injection of TI | 410·2±80·7 | |

*Effect of TI on the rate of cell division*

When cells were removed from TI-treated and control mice, washed and their *in vitro* rates of $^{14}C$ labelled thymidine uptake compared, no significant differences were detected. Since this result might have been influenced by the absence of TI from the incubation medium, we resorted to *in vitro* preincubation of untreated cells with TI before labelling with thymidine. Again, however, no statistical differences were detected although the experiment was repeated a number of times. We therefore examined the doubling time of the cell population *in vivo*. For this purpose a group of tumour cells was labelled *in vivo* with thymidine over a 2-day growth period. These cells were then recovered and reinjected into 2 groups of mice, one of which received a single dose of TI on the following day. The cells were harvested on the third day after injection and the TCA-precipitable radioactivity per cell was calculated. There was a significant difference in the level of labelling of the two cell populations (Table III). Assuming that growth was exponential, cells in the untreated control mice underwent 4

FIG. 6.—Scanning EM micrograph of the internal surface of mouse abdominal wall 21 hours after the injection of $1 \times 10^8$ EAT cells. There are virtually no cells adhering to the surface. $\times$ approx. 400.

FIG. 7.—The internal abdominal surface of a mouse injected 21 hours previously with $1 \times 10^7$ tumour cells, followed immediately by 30 mg of TI. The tumour cells have adhered to the host's tissue as a monolayer. $\times$ approx. 400.

TABLE III.—$^{3}$*H-thymidine Levels in Prelabelled EAT Cells after* 72 *Hours* in vivo. *The Level of Labelling is Expressed as the TCA-precipitable Radioactivity per* 1 × 10$^{7}$ *Cells*

| | d/min ± s.e. mean | *P* |
|---|---|---|
| Initial level (0 hour) | 35220 ± 7030 | |
| After 72 hours in control mice | 2410 ± 320 | < 0·001 |
| After 72 hours in TI-treated mice | 940 ± 220 | |

cell divisions compared with 5 in the TI-treated group; this represents doubling times of 18 and 14 hours respectively.

*Toxicity of TI to EAT cells*

Since the smaller number of cells recovered from TI-treated tumours was not the result of a slower growth rate, we examined the possibility that it was due to cytotoxicity. As measured by trypan blue dye exclusion, there were no more dead cells present in the TI-treated populations than in the controls (Table IV). This observation assumes that dead

TABLE IV.—*Viability of EAT Cells Recovered from Mice after* 8 *Days of Growth, Using the Trypan Blue Dye Exclusion Test. Viability is Expressed as the Percentage of Cells Excluding the Dye*

| | Viability ± s.e. mean | *P* |
|---|---|---|
| Cells from untreated controls | 98·4 ± 0·9 | > 0·2 |
| Cells from TI-treated animals | 99·1 ± 0·3 | |

cells persist long enough for them to be present in detectable numbers; we therefore monitored the release of $^{51}$Cr from prelabelled cells during *in vitro* incubation with TI and compared this with control cells. There was no detectable difference in the rates of release from the 2 groups (Table V). The evidence suggests, therefore, that at the concentrations used TI is nontoxic to EAT cells.

*Scanning electron microscope observations*

The experiments described above show that the observed reduction in the number

TABLE V.—*Release of* $^{51}$*Cr from Prelabelled EAT Cells after Incubation for* 3 *Hours* in vitro. *Total Releasable Radioactivity is Expressed as the Sum of Counts Released into the Supernatant during Incubation Plus Additional Radioactivity Released when Cells were Heat Killed*

| | Total releasable (ct/min ± s.e. mean) | Released (ct/min ± s.e. mean) | *P* |
|---|---|---|---|
| TI added to medium at 5 mg/ml | 1850 ± 91 | 465 ± 23 | > 0·3 |
| Control | 1725 ± 108 | 541 ± 22 | |

of recoverable tumour cells from TI-treated animals could not be explained by a reduction in the total tumour cell population. We therefore examined the internal abdominal surfaces of TI-treated and control mice to see if tumour cells were being retained by the host as the result of injections of TI. When the ventral abdominal wall was examined we found, 21 hours after tumour injection, that virtually no cells adhered to this surface in control animals (Fig. 6), even when 1 × 10$^{8}$ cells were injected. However, in animals which received 30 mg of TI immediately after the injection of tumour cells, large numbers of the cells adhered to the host's tissues (Fig. 7). The cells appeared to form a monolayer; in no instance did we recognize ascites cells adhering to each other. Adherent cells were characterized by processes extending from their basal surface and ramifying over the adjacent host surface.

### DISCUSSION

*Effect of TI on the number and size of recoverable tumour cells*

Groups of mice injected i.p. with EAT cells and subsequently i.p. with either TI in saline or saline alone on alternate days, were compared in respect of the total number of recoverable cells at different periods over the next 10 days. There was consistently a statistically significant reduction in the yield from the TI-treated groups (Fig. 1–5). Cell size distribution analysis indicated that this reduction was not attributable to tumour cells aggregating *in vivo*. Even in groups of mice killed on Day 2 after a single injection of TI (Fig. 3) there was a reduction of 75% in the number of recoverable cells. This reduction was not attributable to a TI-induced initial lag phase in the growth rate, since the mean rate of cell division during the first 72 hours following transplantation was significantly greater in TI-treated than in control cell populations (Table III).

There was also a significant difference in the mean size of control cells (700–800 $\mu m^3$) compared with TI-treated cells (400–600 $\mu m^3$). After 2 days of tumour growth and a single injection of TI, the treated group showed a single peak at 270 $\mu m^3$, whereas the untreated control cells showed 2 peaks (Fig. 3). By Day 4 the controls peaked only at 670 $\mu m^3$ whereas the treated group persisted at 270 $\mu m^3$ (Fig. 5) and this subsequently remained unchanged. When such cells were injected in equal amounts into fresh mice they produced recoverable cell populations which were identical both in respect of total number and mean size, irrespective of whether they originated from TI-treated or control mice (Fig. 4a,b). Thus the 2 populations are composed of similar cells, the difference in size being attributable to the action of TI. This difference in size is not, however, clearly correlated with a change in DNA content (Table I). It cannot therefore be attributable to the change in size which is seen in EAT and other tumour cells as a consequence of the reduced ploidy resulting from the increased growth rate which follows tumour transplantation (Basleer and Desaive, 1971; Dombernowsky, Bichel and Hartmann, 1973). It must therefore be due to a decrease in the mean volume of cytoplasm, probably as the result of the increased growth rate of TI-treated cells.

*Effect of TI on the growth rate of tumour cells*

It seemed reasonable to suppose that the considerable reduction in the number of recoverable tumour cells produced by treatment with TI was the result of a reduced growth rate. However, when such cells were removed from treated mice and compared with untreated control cells in respect of their relative rates of labelled thymidine incorporation *in vitro*, no difference between the 2 groups could be detected. This did not, however, eliminate the possibility that the cells might rapidly revert to the normal rate of growth if TI was removed from the medium or from the cell surface during the washing and dilution procedures associated with this series of experiments. We therefore modified the design, using hitherto untreated tumour cells which were preincubated *in vitro* with TI before comparing the rate of thymidine incorporation with that of untreated control cells. Again, however, no significant differences were observed. In order to reflect more accurately the conditions *in vivo*, we used a population of cells whose DNA had been prelabelled with $^3H$-thymidine, to observe the decline in the level of labelling per cell when grown in TI-treated or control mice. Cells recovered from TI-treated mice contained a significantly lower level of label and were therefore undergoing mitosis at more frequent intervals (Table III). Since analysis of tumour cell kinetics in control mice suggested that growth is almost exponential for the first 10 days after the injection of $1 \times 10^6$ tumour cells, the figures in Table III indicate recoverable cell population doubling times of 14 hours for TI-treated and 18 hours for control cells. This clearly indicates that TI has no inhibitory effect upon mitotic rate.

*Toxicity of TI*

The fact that the viability of TI-treated cells, as judged by the exclusion of trypan blue dye, was the same as that of controls (Table IV) does not entirely exclude the possibility that TI was toxic to the cells, unless we assume that dead cells persist for a sufficient period to represent a statistically detectable proportion of the total population. This problem was eliminated by the observation that cells preincubated in $^{51}Cr$ released label at the same rate when incubated *in vitro*, whether or not TI was added to the incubation medium (Table V). On the basis of these observations, it is apparent that TI does not kill or lyse cells at the concentrations used and the reduction in the number of cells recovered from TI-treated mice cannot be attributed to such an effect.

*Adhesion of tumour cells to host tissues*

Our line of EAT cells does not normally adhere to the internal abdominal surfaces of the host in which it is grown (Fig. 6). Consequently, the mean number of cells recoverable from untreated mice accurately reflects the actual tumour size. When such tumours were treated with TI the number of cells subsequently recovered was markedly reduced when compared with controls. This was due to large numbers of cells adhering to the host's tissues as a result of TI treatment (Fig. 7) and not to any cytotoxic effect or alteration in growth rate. Direct evidence that the adhering cells are indeed tumour cells is being prepared at the moment, but present evidence indicates that they are not inflammatory cells. Cells from TI-treated and control animals formed a single homogeneous population (Fig. 4a,b), which would not be the case if a substantial number of cells from the TI-treated mice were peritoneal macrophages. Furthermore, the normal peritoneal cell population of non tumour-bearing mice was unaltered 6 hours after TI injection (Table II). Consequently, it is unlikely that there is an inflammatory response to TI, and thus the cells adhering to the abdominal wall are most probably tumour cells.

Whether or not such adherent cells were undergoing subsequent cell divisions has not yet been established. The fact that they appeared to form a tightly packed monolayer (Fig. 7) might suggest that further growth had been arrested by contact inhibition, manifested as a result of the TI mediated cell surface change which caused them to adhere to host tissue. However, contact inhibition is normally associated with cells grown on impenetrable supports; in the present case it is possible that cells invade the underlying host tissues, and the appearance of an apparently well ordered monolayer may be deceptive.

The fact that TI-treated cells adhere to host tissues is of considerable theoretical interest and there are a number of possible explanations to account for it. TI may act as an agglutinin by forming cross-linkages between cells which result in aggregation (Inbar and Sachs, 1969). However, it would have to possess two binding sites of different specificities in order to account for its observed failure to agglutinate the EAT cells at the same time as it promoted their binding to host cells. Furthermore, although Con A binds in similar amounts to the membranes of normal and malignant cells (Ozanne and Sambrook, 1971) it does not initiate agglutination in untransformed cells unless the cell surface binding sites are reorientated by proteolytic activity (Nicolson, 1972). Consequently, if TI forms bridges between tumour cells and host cells it is also necessary to postulate that the host cell binding sites are exposed in the " reorientated " manner under normal conditions. For these reasons it appears extremely unlikely that TI mediates the adhesion of tumour and host cells by such a mechanism.

A more likely possibility is that TI alters the surface of either the host or the tumour cell in such a way as to complete a system of binding sites directly linking the two cell types. This linkage would have a degree of specificity to account for the failure of tumour cells to bind to each other. The presence of TI may allow the build up of specific binding sites on the tumour cell surface, by inhibiting an intrinsic protease of tumour origin which otherwise destroys the sites. This hypothesis is supported by the demonstration that the agglutination of EAT cells by

Con A is greatly enhanced by preincubation with TI, (Payne, Whur and Robson, 1973). Presumably Con A agglutination sites are increased in the presence of this protease inhibitor, in line with the finding that the agglutination of transformed cells by Con A is reduced after trypsin treatment (Inbar and Sachs, 1969). We postulate, therefore, that EAT cells bind to host tissue as a result of the inhibition by TI of intrinsic proteolytic activity. This hypothesis receives support from observations which indicate that treatment of malignant cells with protease inhibitors *in vitro* results in an alteration of growth characteristics, which may result from modification of the cell surface (Goetz, Weinstein and Roberts, 1972; Schnebli and Burger, 1972). Our findings support the view that the abnormal growth rates and adhesive properties of tumour cells are related to modification of cell surface binding sites by the activity of intrinsic proteases.

We thank Miss P. Murray for technical assistance. We would also like to record our appreciation to Dr D. C. Williams, Head of the Research Department, for his constant interest and encouragement.

### REFERENCES

Basleer, R. & Desaive, C. (1971) Contribution to the Study of the Ehrlich Ascites Tumour Cells. A Cytological and Cytochemical Analysis of the Effects of Sarcolysine. *Eur. J. Cancer*, **7**, 441.

Burger, M. M. (1969) A Difference in the Architecture of the Surface Membrane of Normal and Virally Transformed Cells. *Proc. U.S. Acad. Sci.* **62**, 994.

Burger, M. M. (1970) Proteolytic Enzymes Initiating Cell Division and Escape from Contact Inhibition of Growth. *Nature, Lond.*, **227**, 170.

Burger, M. M. & Noonan, K. D. (1970) Restoration of Normal Growth by Covering of Agglutinin Sites on Tumour Cell Surface. *Nature, Lond.*, **228**, 512.

Burton, K. (1956) A Study of the Conditions and Mechanism of the Diphenylamine Reaction for the Colorimetric Determination of Deoxyribonucleic Acid. *Biochem. J.*, **62**, 315.

Dombernowsky, P., Bichel, P. & Hartmann, N. R. (1973) Cytokinetic Analysis of the JB-1 Ascites Tumour at Different Stages of Growth. *Cell & Tiss. Kinet.*, **6**, 347.

Goetz, I. E., Weinstein, C. & Roberts, E. (1972) Effects of Protease Inhibitors on Growth of Hamster Tumor Cells in Culture. *Cancer Res.*, **32**, 2469.

Inbar, M. & Sachs, L. (1969) Interaction of the Carbohydrate Binding Protein Concanavalin A with Normal and Transformed Cells. *Proc. U.S. Acad. Sci.*, **63**, 1418.

Nicolson, G. L. (1972) Topography of Membrane Concanavalin A Sites Modified by Proteolysis. *Nature, New Biol.*, **239**, 193.

Ozanne, B. & Sambrook, J. (1971) Binding of Radioactively Labelled Concanavalin A and Wheat Germ Agglutinin to Normal and Virus-transformed Cells. *Nature, New Biol.*, **232**, 156.

Payne, N. E., Whur, P. & Robson, R. T. (1973) Agglutination of Normal and Malignant Cells by Concanavalin A in Relation to Cell Surface Structure. In: *Proceedings 14th Annual General Meeting British Association for Cancer Research.* Abstract in *Br. J. Cancer*, **28**, 86.

Pickaver, A. H., Ratcliffe, N. A., Williams, A. E. & Smith, H. (1972) Cytotoxic Effects of Peritoneal Neutrophils on a Syngeneic Rat Tumour. *Nature, New Biol.*, **235**, 186.

Schnebli, H. P. & Burger, M. M. (1972) Selective Inhibition of Growth of Transformed Cells by Protease Inhibitors. *Proc. U.S. Acad. Sci.*, **69**, 3825.

Whur, P. & Weatherhead, B. (1971) Rates of Incorporation of ($^3$H) Leucine into Protein of the Pars Intermedia of the Pituitary in the Amphibian *Xenopus laevis* after Change of Background Colour. *J. Endocr.*, **51**, 521.

Winzler, R. J. (1970) Carbohydrates in Cell Surfaces. *Int. Rev. Cytol.*, **29**, 77.